# The potential to produce tropodithietic acid by *Phaeobacter inhibens* affects the assembly of microbial biofilm communities in natural seawater

**DOI:** 10.1038/s41522-023-00379-3

**Published:** 2023-03-23

**Authors:** Pernille Kjersgaard Bech, Sheng-Da Zhang, Nathalie Nina Suhr Eiris Henriksen, Mikkel Bentzon-Tilia, Mikael Lenz Strube, Lone Gram

**Affiliations:** Department of Biotechnology and Biomedicine, Technical University of Denmark, Lyngby, Denmark

**Keywords:** Biofilms, Water microbiology

## Abstract

Microbial secondary metabolites play important roles in biotic interactions in microbial communities and yet, we do not understand how these compounds impact the assembly and development of microbial communities. To address the implications of microbial secondary metabolite production on biotic interactions in the assembly of natural seawater microbiomes, we constructed a model system where the assembly of a natural seawater biofilm community was influenced by the addition of the marine biofilm forming *Phaeobacter inhibens* that can produce the antibiotic secondary metabolite tropodithietic acid (TDA), or a mutant incapable of TDA production. Because of the broad antibiotic activity of TDA, we hypothesized that the potential of *P. inhibens* to produce TDA would strongly affect both biofilm and planktonic community assembly patterns. We show that 1.9 % of the microbial composition variance across both environments could be attributed to the presence of WT *P. inhibens*, and especially genera of the Bacteriodetes were increased by the presence of the TDA producer. Moreover, network analysis with inferred putative microbial interactions revealed that *P. inhibens* mainly displayed strong positive associations with genera of the *Flavobacteriaceae* and *Alteromonadaceae*, and that *P. inhibens* acts as a keystone OTU in the biofilm exclusively due to its potential to produce TDA. Our results demonstrate the potential impact of microbial secondary metabolites on microbial interactions and assembly dynamics of complex microbial communities.

## Introduction

Complex and dynamic microbial communities colonize nearly every ecosystem in marine environments, and major biogeochemical processes, such as carbon cycling, are largely driven by microorganisms^1^. Both stochastic and deterministic abiotic and biotic processes can affect the assembly and composition of microbial communities^2^. Abiotic factors such as temperature and resource availability have a major influence on the taxonomic composition of microbiomes^3,4^, but inter-kingdom and interspecific interactions between co-occurring organisms may play equally important roles^5,6^. Competitive as well as cooperative interactions within microenvironments may be mediated by the production of secondary (or ‘specialized’) metabolites produced by a multitude of microorganisms^7–9^. These metabolites have predominantly been exploited because of their therapeutic, and agri- and aqua-cultural benefits. Studies on their potential ecological role(s) have generally described them as mediators of interference competition between microbes^10^, and most experiments have been conducted in co-cultures, or as traditional antibiotic screening assays^11,12^. The perception that microbial interactions are primarily negative has however recently been challenged as growth-promoting positive interactions have proven prevalent among soil community members^13^.

Mechanistic understanding of how microbial secondary metabolite production drives the assembly of natural microbial communities remains limited, largely due to the methodological challenge of (re)creating complex natural environmental conditions in the laboratory. The introduction of cell-free extracts containing secondary metabolites can either reduce or enrich specific taxa in semi-natural mesocosm communities^14^. While the addition of defined concentrations of pure compounds resulting in a homogenous suspension may resemble the planktonic environmental conditions of free-living bacteria, it is far from conditions found in bacterial biofilm. The surface associated bacteria in biofilms live in spatially structured communities, in which microscale gradients occur due to the consumption of resources and secretion of secondary metabolites^15,16^. In this study, we constructed a model system allowing for the formation of such spatially structured communities and introduced a bacterial producer of a potent antibiotic secondary metabolite.

The *Rhodobacterales* are ubiquitous in the marine environment, where in particular the *Roseobacter* group such as *Tritonibacter* and *Phaeobacter* spp. are primary colonizers on freshly submerged surfaces^17^. While the *Tritonibacter* genus (formerly *Rugeria*) have been isolated from the free-living planktonic phase^18^ the genus *Phaeobacter* has typically been isolated from marine biofilms^19,20^. Also, *Phaeobacter* are capable of invading preformed bacterial biofilms^21–23^. Several *Phaeobacter* spp. can produce the antibacterial secondary metabolite tropodithietic acid (TDA)^24,25^, and previous studies have suggested that TDA has multiple additional functions, such as being a weak iron chelator and having hormetic effects on nearby organisms. Thus, TDA may act as a mediator of antibiosis, or interspecific signaling in sub-inhibitory concentrations^26,27^.

To determine how such a secondary metabolite producer affects biofilm formation and the proximate planktonic microbiome, we introduced a wild type and a mutant incapable of producing this particular metabolite, respectively, into our model systems. Thus, the TDA producing *P. inhibens* and the TDA deficient mutant were separately allowed to colonize abiotic surfaces that were then submerged in natural seawater. Here, we hypothesized that the wildtype *Phaeobacter*, capable of TDA production, would cause changes in the microbiome composition during assembly of both environments potentially eliminating specific taxonomic groups. Deciphering such changes will provide insights into how microbial secondary metabolites can mediate ecological microbial community associations.

## Results

A semi-natural marine microbial model system was constructed to determine if, and how, the potential of a bacterium to produce a potent secondary metabolite affected biofilm formation. The WT and the dTDA *P. inhibens* strains were allowed to pre-form biofilms and the subsequent colonization and change in microbial composition in a natural seawater system was studied and compared to changes in a system without pre-colonization. Since metabolic interactions change over time, we sampled over a 10-day period. To investigate the potential production of TDA in the axenic model system with the WT, we constructed a GFP reporter fusion in the *P. inhibens* DMS17935 strain to track expression from the *tdaCDE* promoter as a proxy for TDA production. Qualitative GFP fluorescence detection was observed after day 0, 1, 4 an 10 days, indicating continuous transcription of the *tdaCDE* operon and suggesting potential production of TDA in this system (Supplementary Fig. 1).

### Quantification of absolute microbial abundances and *Phaeobacter* colonization

**Fig. 1:**
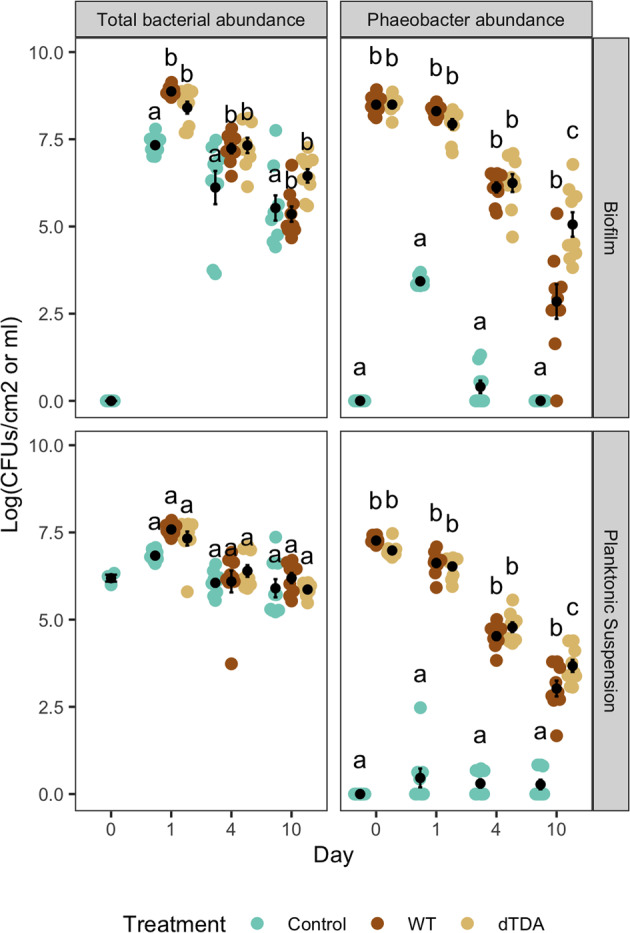
Total cell counts. The absolute abundance of the total microbial population and *Phaeobacter* spp. across all samples was estimated by qPCR and standardized to log_10_ transformed CFUs/cm or mL found in the microbial biofilm or in the planktonic suspension, respectively. Shown in black are means (dots) and standard deviation (error bars) and significance letters (LMM & EMM; *p-*value < 0.05) between the treatments per day. Source data are provided as a Source Data file.

In the non-treated system, the biofilm bacterial community from natural seawater reached 7.33 ± 0.3 log_10_ CFU/cm^2^ after one day. This was significantly lower than the biofilm level of 8.87 ± 0.1 and 8.41 ± 0.5 log_10_ CFU/cm^2^ in the WT and the dTDA (LMM & EMM; *p-*value < 0.05), respectively. After the first day, the cell densities across all treatments gradually decreased over time. Both the WT and the dTDA treated systems had similar and non-significantly different total abundances up until day 4 (LMM & EMM; *p-*value = 1.00). At day 10, the total bacterial abundances of the WT treated system had significantly decreased 1 log_10_ fold compared to the dTDA system (LMM & EMM; *p-*value = 0.03) and was similar to the control treatment corresponding to 5.36 ± 0.7 log_10_ CFU/cm^2^ (Table 1; Fig. 1).

**Table 1:** Levels of *Phaeobacter inhibens* in the three treatments over time and the total bacterial abundance dertermined by qPCR given as log_10_ (CFU/cm^2^ or CFU/mL) value.

Sample type	Treatment	Day	n	*P. inhibens* relative abundance (%)	*P. inhibens*	Total abundance	Observed richness
					Log_10_ (CFU/cm^2^ or /mL)		
Planktonic suspension	Native	0	3	0.02 ± 0.01	no *ct*	6.19 ± 0.2	331 ± 18
Biofilm	Control	1	9	0.04 ± 0.06	3.43 ± 0.1	7.33 ± 0.3	253 ± 70
		4	9	0.00 ± 0.02	0.40 ± 0.5	6.12 ± 1.4	235 ± 55
		10	9	0.00 ± 1.18	0.00 ± 0.0	5.53 ± 1.1	209 ± 42
	WT	1	9	80.11 ± 26.86	8.31 ± 0.2	8.87 ± 0.1	75 ± 47
		4	9	12.65 ± 5.13	6.12 ± 0.4	7.23 ± 0.4	215 ± 56
		10	9	1.25 ± 7.52	2.85 ± 1.5	5.36 ± 0.7	208 ± 75
	dTDA	1	8	66.10 ± 13.74	7.93 ± 0.4	8.41 ± 0.5	107 ± 54
		4	8	16.11 ± 12.65	6.25 ± 0.8	7.32 ± 0.7	202 ± 59
		10	9	1.58 ± 5.72	5.06 ± 1.1	6.45 ± 0.6	219 ± 39
Planktonic suspension	Control	1	9	0.00 ± 0.01	0.46 ± 0.8	6.83 ± 0.2	206 ± 32
		4	9	0.00 ± 0.03	0.30 ± 0.4	6.05 ± 0.3	206 ± 14
		10	9	0.13 ± 2.68	0.27 ± 0.4	5.90 ± 0.8	122 ± 60
	WT	1	9	58.33 ± 14.68	6.62 ± 0.3	7.59 ± 0.2	92 ± 36
		4	8	3.34 ± 0.78	4.52 ± 0.3	6.09 ± 0.9	207 ± 54
		10	9	0.23 ± 0.15	3.02 ± 0.7	6.19 ± 0.4	194 ± 39
	dTDA	1	9	43.05 ± 15.29	6.52 ± 0.3	7.33 ± 0.6	108 ± 35
		4	9	6.05 ± 3.56	4.78 ± 0.4	6.39 ± 0.5	193 ± 53
		10	8	0.64 ± 2.61	3.67 ± 0.5	5.86 ± 0.2	184 ± 58
Biofilm	WT	0	9	100	8.49 ± 0.3	nd^a^	1
	dTDA	0	9		8.49 ± 0.2		
Planktonic suspension	WT	0	9	nd^a^	7.27 ± 0.1	nd^a^	nd^a^
	dTDA	0	9		6.98 ± 0.2		

^a^nd: not determined

The relative abundance of the *Phaeobacter inhibens* OTU based on amplicon sequencing and the observed richness (genus level) based on amplicon sequencing is also provided.

The WT and the dTDA *P. inhibens* strains readily colonized steel surfaces to the same level (LMM & EMM; *p*-value = 1) of 8.49 ± 0.3 and of 8.49 ± 0.2 log_10_ CFU/cm, respectively, before being exposed to the natural sea water (Table 1; Fig. 1).

Even though the surfaces were rinsed, patches of biofilm still detached from the steel coupons upon submersion in seawater and levels of approx. 10^7^ CFU/mL of the WT and dTDA were detected in the planktonic suspensions. After one day, the WT and dTDA were reduced to 8.31 ± 0.2 and 7.93 ± 0.4 log_10_ CFU/cm in the biofilm (LMM & EMM; *p*-value=0.951) and to 6.2 ± 0.3 and 6.52 ± 0.3 log_10_ CFU/mL in the seawater (LMM & EMM; *p*-value = 1). *Phaeobacter* abundances continued to decrease after four days in all systems; in the biofilm to 6.12 ± 0.4 and 6.25 ± 0.8 log_10_ CFU/cm (LMM & EMM; *p*-value = 1) and in the planktonic suspension 4.52 ± 0.3 and 4.78 ± 0.4 log_10_ CFU/mL (LMM & EMM; *p*-value = 0.829). Numbers decreased further after ten days of incubation with a significant additional decrease in the numbers of WT as compared to the dTDA and 2.85 ± 1.5 and 5.06 ± 1.1 log_10_ CFU/cm^2^ were detected in the biofilm (LMM & EMM; *p*-value=0.0001) and 3.02 ± 0.7 and 3.67 ± 0.5 log_10_ CFU/mL in the planktonic suspension (LMM & EMM; *p*-value = 0.029). No *Phaeobacter* spp. were detected by the qPCR in the natural seawater (No ct values), however, a short-term *Phaeobacter* spp. colonization of stainless steel was observed at day one, corresponding to 3.43 ± 0.1 log_10_ CFU/cm, yet significantly lower as compared to the inoculated *Phaeobacter* spp. abundances (*p-*value < 0.001). The remaining numbers in the Control across all days and treatments were < 0.5 log_10_ CFU/cm. As expected, the relative abundance of *Phaoebacter* (WT and dTDA) determined from the amplicon sequencing was high in samples pre-inoculated with the strains (Supplementary Fig. 2B).

### Factors driving the microbial community composition

We assumed that *time* (day 1, 4, or 10), *treatment* (Control, dTDA or WT systems) and *environment* (biofilm or planktonic suspension) would predict the microbial community assembly. The *P. inhibens* OTU were removed from the analyses to exclude the variance caused by its high relative abundance. The beta-dispersion was non-significant (*p*-value > 0.05) and similar across all experimental combinations.

**Fig. 2:**
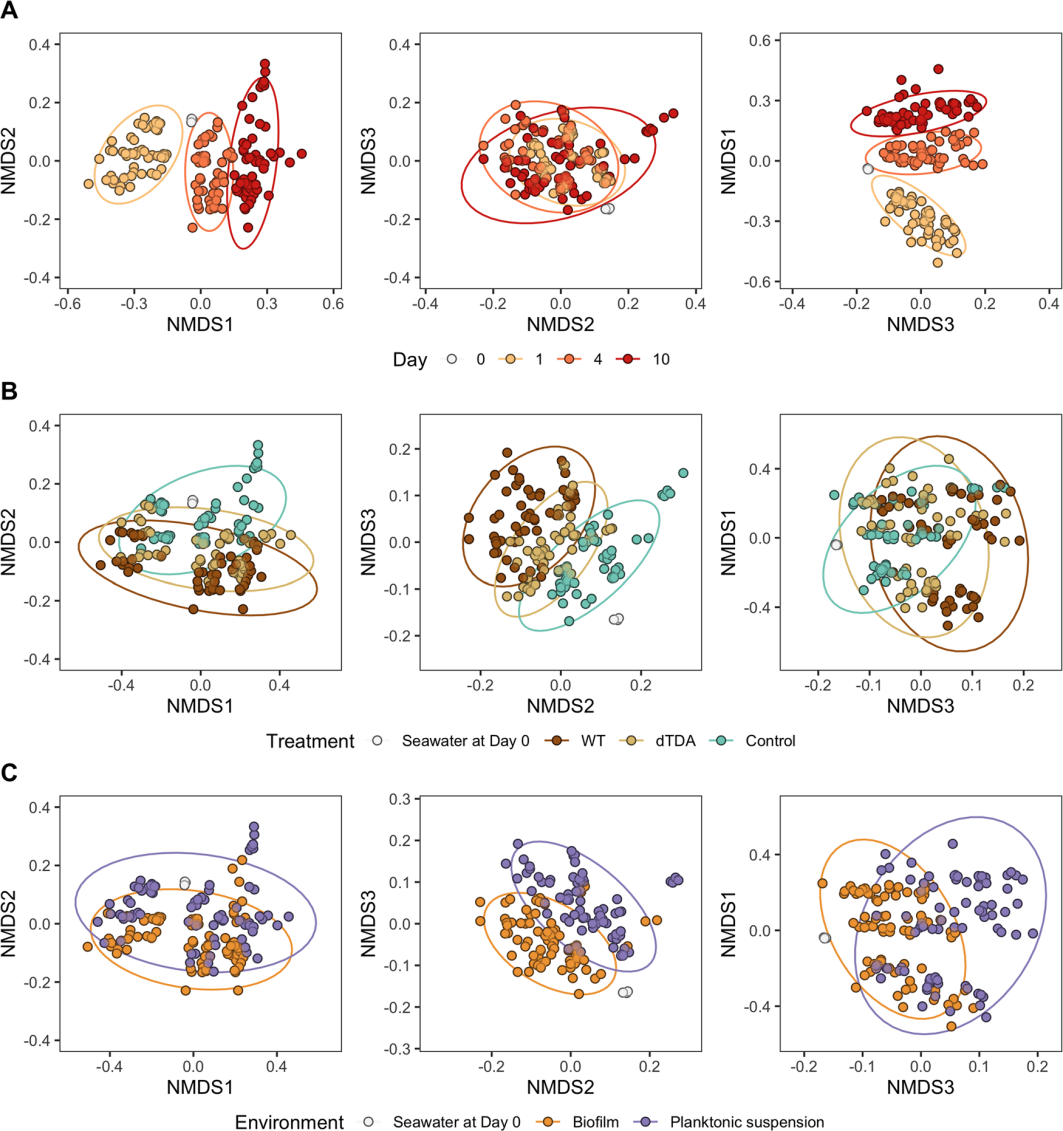
Microbial community composition. NMDS ordination plot of V3-V4 16S gene amplicon data from marine biofilm and planktonic communities treated with or without the WT and the dTDA *P. inhibens* using Bray*-*curtis distances (k = 3, stress = 0.087, non-metric fit, R2 = 0.993). Samples are stratified by **A** Time, **B** Treatment and **C** Environment. Source data are provided as a Source Data file.

Multivariate analysis using PERMANOVA revealed that 76.1 % of the variation in the microbial composition across the full data set was explained either individually or in combinations of time, environment and treatment (Table 2). Time was the main factor driving diversity in the bacterial communities (R^2^ = 0. 417, *p-*value = 0.001), followed by the treatment (R^2^ = 0. 122, *p-*value = 0.001) although the variance explained by the treatment was dependent on time (R^2^ = 0. 091, *p-*value = 0.001). Differences were also dictated by the environment *e.g*. the compositional variance between biofilm compared to the planktonic suspension (R^2^ = 0.059, *p-*value = 0.001) and this effect was dependent on time (R^2^ = 0.041, *p-*value = 0.001) and treatment (R^2^ = 0.014, *p-*value = 0.001). Multilevel pairwise PERMANOVA between the WT and dTDA systems furthermore showed that community composition was directly influenced by the potential of *P. inhibens* to produce TDA (Table 2; R^2^ = 0. 019, *p-*value = 0.001), although this effect was significantly mediated by interaction with time (R^2^ = 0.015, *p-*value = 0.001). The significant conditions driving community assembly were further supported by the clustering in nMDS ordination plot (Fig. 2).

**Table 2:** Results of PERMANOVA and a reduced model using multilevel pairwise PERMANOVA (pairwise.adonis2) on independent variables and their interactions driving the microbial community composition from all samples combined (genus level). Distances are Bray-Curtis. *Phaeobacter inhibens* OTU was excluded.

	PERMANOVA			Multilevel pairwise PERMANOVA					
				WT vs. Control		WT vs. dTDA		dTDA vs. Control	
Conditions	F	R2	*p*-value	R2	*p*-value	R2	*p*-value	R2	*p*-value
Treatment	24.210	0.122	0.001	0.061	0.001	0.019	0.001	0.021	0.001
Environment	35.077	0.059	0.001	0.034	0.001	0.029	0.001	0.049	0.001
Day	123.908	0.417	0.001	0.709	0.001	0.815	0.001	0.763	0.001
Treatment:Environment	4.141	0.014	0.001	0.004	0.013	0.002	0.146	0.003	0.076
Treatment:Day	13.553	0.091	0.001	0.013	0.001	0.015	0.001	0.023	0.001
Environment:Day	12.157	0.041	0.001	0.082	0.001	0.021	0.001	0.040	0.001
Treatment:Environment:Day	2.477	0.017	0.001	0.007	0.006	0.005	0.047	0.006	0.023

### *Phaeobacter* pre-colonization reduces the microbial community complexity

Alpha diversity was determined as the total sum of observed unique genera across all samples. The richness decreased significantly in all systems as compared to the natural seawater samples (Dunnest test; *p*-values < 0.01; Table 1; Supplementary Fig. 2A). Only the Control system on day 4 had a complexity similar to the natural seawater (Dunnest test; *p*-value = 0.3061). After the first day of incubation, the difference between the observed richness of the WT and dTDA systems in the biofilm was non-significant, comprising 75 ± 47 and 107 ± 54 unique genera, yet both were substantially lower than the complexity of the Control system 253 ± 18 (LMM & EMM; *p*-values < 0.001). A similar pattern was seen for the planktonic suspension of the WT and dTDA systems, where the observed richness was reduced to 92 ± 36 and 108 ± 35 unique genera compared to the Control system 206 ± 32 (LMM & EMM; *p*-values < 0.001), respectively. The complexity remained unchanged and non-significant between treatments on day 4 and day 10 in both environments (LMM & EMM; *p*-values > 0.05).

### Identification of genera that solely respond to the potential TDA production by *Phaeobacter inhibens*

**Fig. 3:**
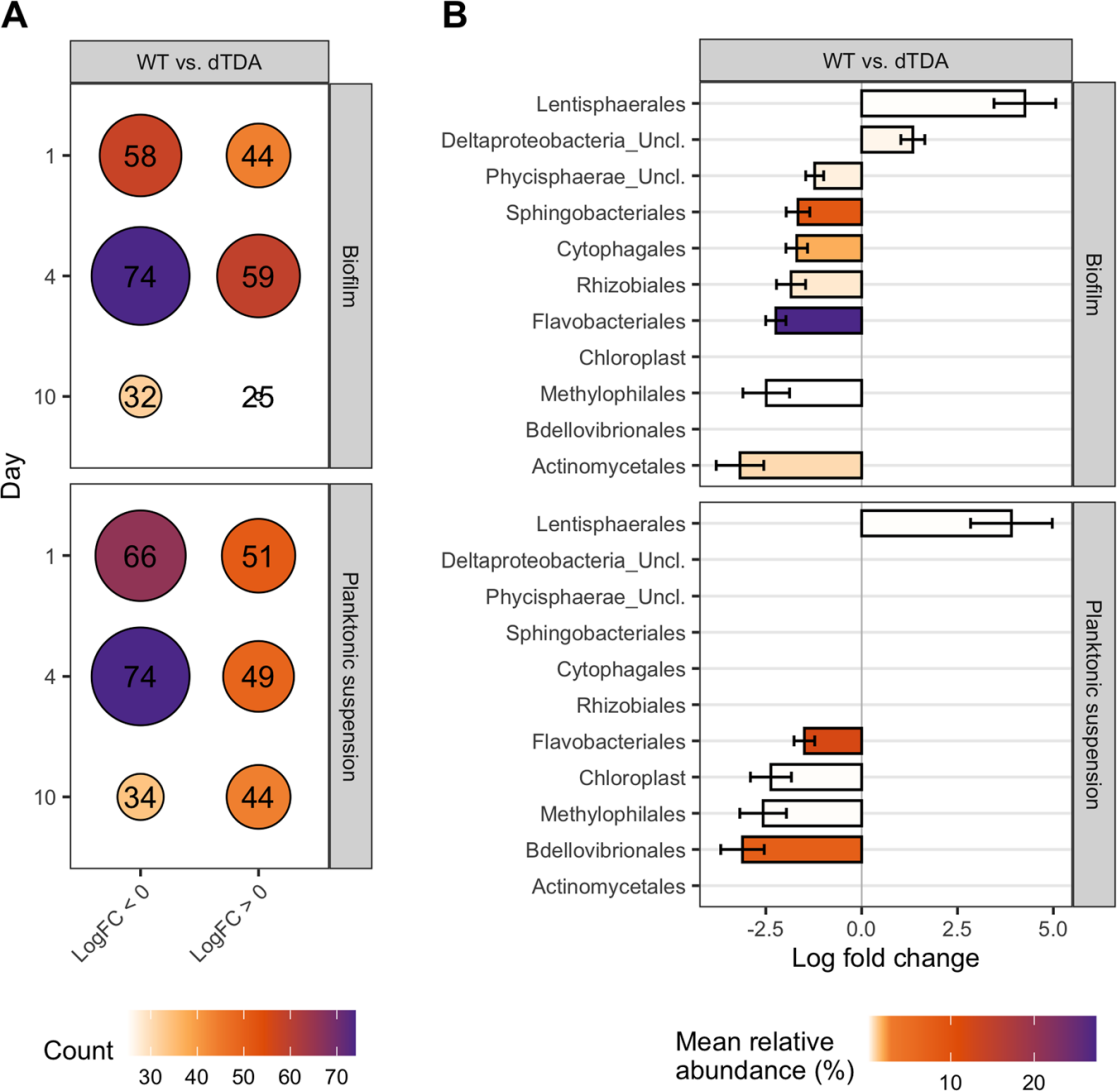
Identification of taxa that were differentially abundant between the WT and the dTDA systems. Taxa were identified by the ANCOM-BC model using a 95 % confidence interval (two-sided; Holm adjusted) and adjusted *p*-values. **A** Bubbles representing the size equivalent to the accumulated sum of unique genera that are enriched in the respective treatment relative to the other. **B** ASVs are aggregated to order level and represented by effect size (Log_10_ fold change). Bars are coloured according to the mean relative abundance of the order enriched by the treatment relative to the other. Source data are provided as a Source Data file.

We used the ANCOM-BC model to analyse differential relative abundance between the WT versus the dTDA treated systems stratified by day and environment. Of 1056 different genera found in the total dataset, 219 unique genera were increased in their relative abundances by the WT and 176 genera were reduced relative to the dTDA system over the 10-day period in both environments. Because the largest sum of differentially abundant genera was observed at day 4, we assumed that this was also the day on which the presence of the TDA producing wildtype had the most marked effect (Fig. 3A). Therefore, we re-analyzed the differential abundance in all three combinations at order level to get an overall view on the taxonomic profile from day 4 (Fig. 3B).

Orders that were specifically increased by the WT were the *Flavobacteriales* (27.4 %), *Sphingobacteriales* (8.8 %), *Cytophagales* (1.8 %), *Actinomycetales* (0.9 %), *Rhizobiales* (0.6 %), Phycisphaerae (0.4 %), and *Methylophilales* (0.1 %), whereas only *Lentisphaerales* (0.2 %), while only an unclassified order of Deltaproteobacteria (0.6 %) were increased by the dTDA strain in the biofilm. In the planktonic suspension, again the *Flavobacteriales* (11.9 %) were increased in the presence of the WT compared to dTDA strain, together with the *Bdellovibrionales* (7.5 %), Chloroplast (0.2 %) and *Methylophilales* (0.1 %). Several Flavobacteria were significantly increased in the WT treated system in both environments compared to both the dTDA and the control system (Supplementary Fig. 3).

### The TDA-producing *P. inhibens* becomes a keystone OTU in the microbial network in surface colonization

Since the pre-colonization of *P. inhibens* had a marked effect on microbial community composition, we used network analysis to provide insight into the microbial community structure and to disentangle whether the *P. inhibens* OTU abundances were directly co-occurring with other genera that also increased in relative abundance in response to the presence of the WT. Twelve paired networks were separately constructed for each day and environment with the network comparison module of NetCoMi, since PERMANOVA and ANCOM-BC analysis revealed that the impact of TDA-producing *Phaeobacter* depends on its spatial organization and time. For each pairwise network comparison, we compared two network properties that have been associated with stability of ecological community studies: (1) the positive edge percentage and (2) the modularity^28–30^. Next, we identified hubs or ‘keystone genera’ among common nodes between the WT and dTDA systems. All network scores for each node are listed in Supplementary Table 1.

The positive edge percentage and the modularity of each network comparison were similar and non-significant between the WT and the dTDA systems across time and each environment, except for the planktonic samples at day 4 and the biofilm at day 10. A weakly significant difference in the modularity of the dTDA system was calculated in the planktonic suspension at day 4 (Permutation tests; *p*-value = 0.012). In contrast, the opposite was found for the modularity of the WT in the biofilm at day 10 (Permutation tests; *p*-value = 0.039), suggesting that neither the WT nor the dTDA had any evident effect on the resilience of the microbiome.

At the first day, both comparison networks were dominated by the most influential nodes being genera of the Bacteroidetes, where in particular genera of the Flavobacteriia were among the most central nodes in both treatments, yet no significant difference was detected between the two treatments.

**Fig. 4:**
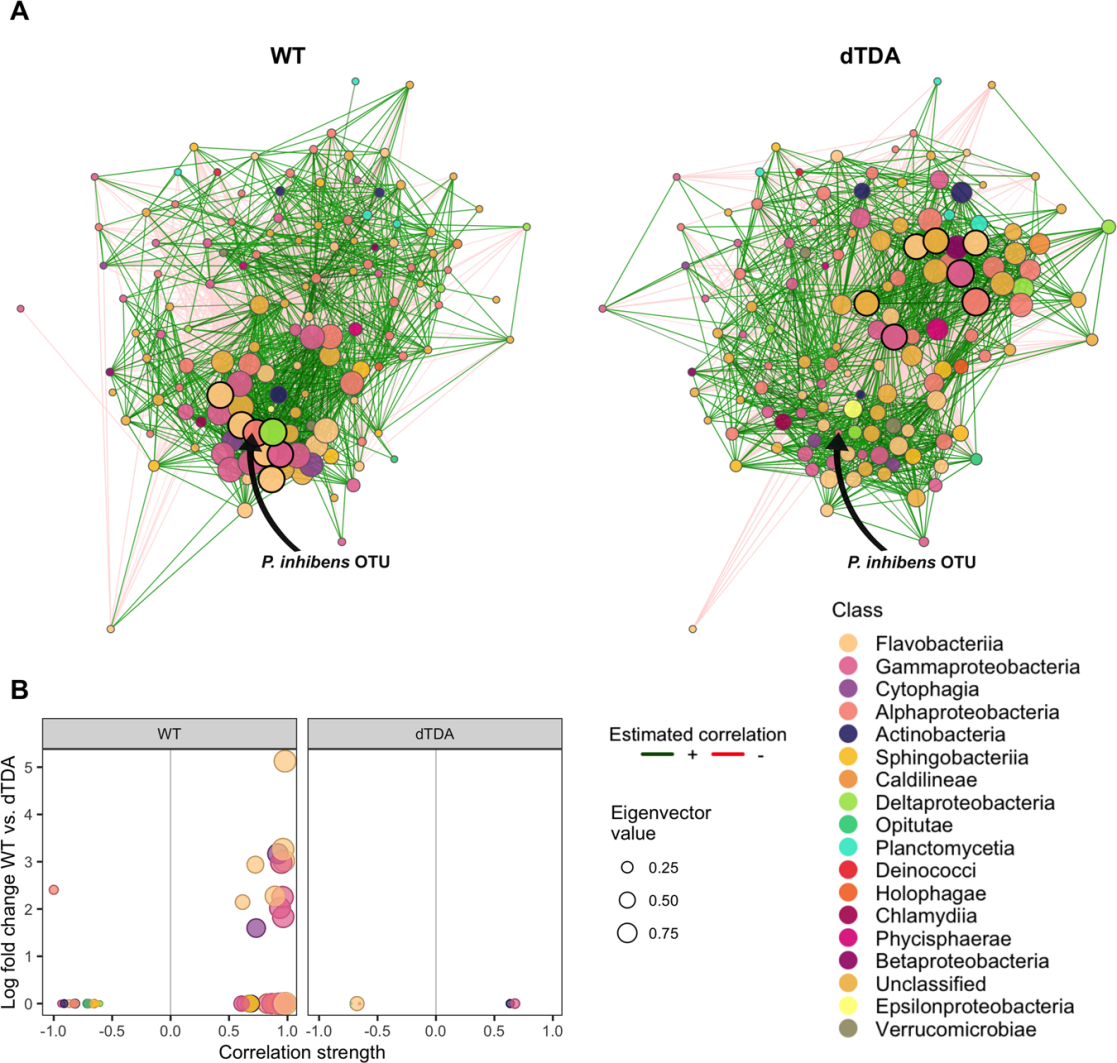
Network analysis of key taxa. **A** Co-occurrence comparison networks between 135 genera (nodes) found in the WT and dTDA treated systems at day 4 in the biofilm samples. Edges represent positive (green) and negative (red) correlations > |0.6| calculated by the SparCC method. Nodes represent ASVs aggregated to genus level together with the *P. inhibens* OTU. Eigenvector centrality of each node was used to define hubs (bold). **B** Genera directly associated to the *P. inhibens* OTU in each treatment plotted as a function of the increase in relative abundance in the WT relative to the dTDA treated systems identified by the ANCOM-BC analysis and the correlation strength found by the SparCC method. Node colors represent the taxonomic class of the genera and sizes were scaled according to their eigenvector centrality score. Source data are provided as a Source Data file.

Interestingly, the WT *P. inhibens* node was identified as a hub, with a significantly higher eigenvector centrality as compared to the dTDA *P. inhibens* OTU (Permutation tests*; p*-value = 0.023) in the biofilm. The WT OTU displayed a high degree of closeness and low betweenness, where 33 of the 55 connections were positively associated with Gammaproteobacteria, Flavobacteriia and Cytophagia genera. All positive edge connections of this node were to other nodes showing high eigenvector centralities as compared to the negative correlations to nodes with very low eigenvector centralities (scores < 0.25; Fig. 4). We could further confirm this by the univariate ANCOM-BC analysis, where we saw the 12 nodes positively connected to the WT *P. inhibens* node were also significantly increased in their relative abundance at day 4 in the biofilm. The WT node also showed a higher eigenvector centrality in the planktonic suspension at day 4 as compared to the dTDA, however this was non-significant. Only few nodes (6 of 135) were connected to the *P. inhibens* dTDA node and what instead seemed to be the most central node was the *Stenotrophomonas*, an opportunistic fish pathogen (Permutation tests*; p*-value = 0.023), which had no connection to the dTDA node. At day 10, no difference was observed between the centrality measures for the WT and dTDA nodes, with the only exception that the dTDA node showed higher closeness centrality to other nodes in the planktonic suspension as compared to the WT (Permutation tests; *p*-value = 0.008).

## Discussion

Microbial secondary metabolites such as antibiotics and siderophores are important mediators of species interactions in many ecological niches, however, whether these secreted metabolites are predominantly weapons of warfare or have other and broader roles being involved in interspecific signaling between species is being discussed^8,31–35^. Nevertheless, it is unknown how such metabolites may mediate microbial community structure and assembly patterns. In this study, we constructed a semi-natural model system to mimic the structural heterogeneity of natural bacterial biofilms. In order to determine the microscale effect on the microbiome assembly by a sequence-based approach, we boosted the system, allowing a naturally occurring biofilm-forming secondary metabolite producer to pre-colonize the system. Thus, we manipulated the microbial assembly patterns in semi-synthetic communities to assess whether the potential to produce the secondary metabolite, TDA, affected biofilm assembly and development. We demonstrate a minor, yet significant, level of explained variance (1.9 %) from the potential of *P. inhibens* to produce TDA across all time points but also observed a significant dependence of time on this effect (1.5 %) in agreement with the most pronounced effect seen at day 4 where *P. inhibens* temporarily became a keystone species solely through its potential to produce TDA. Eventhough TDA was below detection limit in the chemical bulk-extractions, we captured GFP flouresence indicating transcription activity of genes responsible for the TDA biosynthesis in the GFP-reporter fusion strain, suggesting that TDA could be produced when *P. inhibens* is established in the biofilm and the surface associated community.

The semi-natural model system likely experienced nutrient depletion over time, and this may explain the overall decrease of the surface colonizing and free-living *Phaeobacter* spp. in the treated systems, and the increase of relative abundances of autotrophic taxa. The reduction of both *Phaeobacter* strains could further reflect the transition from the initial inoculated abundances going towards a community composition equilibrium as previously described for similar systems^36,37^.

Majzoub et al. ^38^ compared the phenotypic traits between a *P. inhibens* strain with the capability to produce TDA and mutants with mutations in the TDA gene cluster, and showed that biofilm persistence was independent of the capability to produce TDA. Consistent with this study, no difference was observed between the total abundance of the WT and dTDA strain in the beginning. Yet, the significant decrease of the WT compared to the dTDA strain during the last time point at day 10 suggests that the production of TDA and tolerance maintenance may become a metabolic burden for *P. inhibens*.

Time was the main factor influencing microbial community assembly and this, combined with the presumed decrease in nutrient availability, reflects that stochastic drift and genetic diversification are inherent properties of time-dependent ecological studies^2,6^. Nonetheless, the potential to produce TDA had a significant impact on the assembly of the microbial community composition. In some *Roseobacter* group species, TDA biosynthesis is most prominent under static growth conditions^39^; however, *P. inhibens* DSM17935 also produces TDA under shaking conditions^40^, which may explain the significant change in the planktonic suspension under the assumption that TDA is being produced during the course of the experiment.

The present study specifically aimed to test the potential role of the presence of a secondary metabolite producer in community assembly, and given that TDA affect a wide range of microorganisms^25,41^, it was surprising that most genera were increased by the WT but not the dTDA strain. The largest differential changes were observed on day four, where members of the Bacteroidetes, *i.e*. the *Flavobacteriales*, *Sphingobacteriales* and *Cytophagales* were all significantly enriched by the WT compared to the dTDA systems. The positive association between the *P. inhibens* when it is capable of producing TDA and the *Flavobacteriales* including *Winogradskyella* spp. has been observed in previous studies describing different marine systems such as non-axenic algal and oyster microbiomes^42,43^. The fact that several members of the Bacteroidetes are motile by gliding^39^ and that the TDA-producing *Phaeobacter* species most frequently have been isolated from harbor surfaces^20,44^, suggests that they could compete for surface colonization space in nature. However, the role of TDA as a metabolite broadly facilitating interference competition seems unlikely between *P. inhibens* and genera of the Bacteriodetes considering the positive effect of the potential TDA production.

**Fig. 5:**
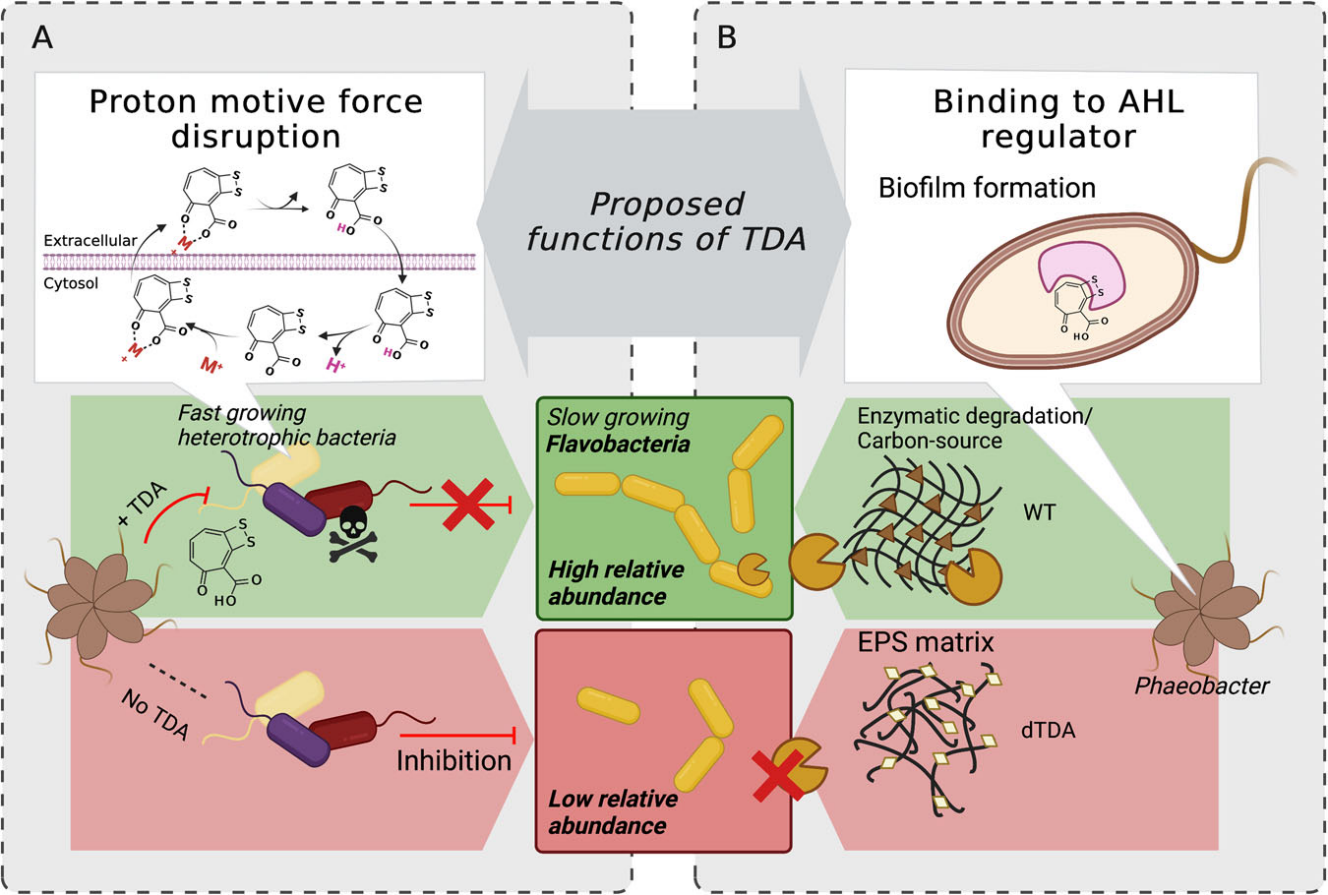
Hypothetical model illustrating the effects of *P. inhibens* and TDA in modulating microbial community assembly. Proposed functions of TDA as reviewed in Henriksen & Lindqvist et al. (2021)^78^ and suggestions for the direct or indirect increase of the relative abundance of different classes such as the Flavobacteria observed in the WT system compared to the dTDA system. **A** The mode of action of TDA has been proposed to disrupt the proton motive force in fast-growing heterotropic bacteria leading to the inhibition of these members of the community that otherwise would outcompete other slow growing bacteria and/or **B** Since TDA has the potential to bind to the AHL regulator that induces changes in motility and biofilm formation^38^, mutations in the TDA gene cluster might affect the extracellular polymeric substance (EPS) composition of the dTDA strain. Consequently, Flavobacteria and other members of the Bacteroidetes might be able to exploit specific compositional elements of the WT EPS matrix as carbon source that are not present in the dTDA EPS matrix. The illustration was created in Biorender.com.

Whether the TDA production was directly involved in an actual facilitation of the increase in these different orders is difficult to ascertain, and a variety of mechanisms could explain the direct or indirect increase of these. Members of the Bacteroidetes are proficient biopolymer degraders^45,46^ and the increase of Bacteroidetes including Flavobacteria spp. could be caused by their capability to exploit the biofilm matrix of the WT as opposed to dTDA strain, as mutations in the TDA gene cluster affect extracellular polymeric substance composition^38^. TDA or the producing bacteria typically have a reducing effect on fast-growing heterotrophic bacteria and we also observed a significant reduction of several members of the Gammaproteobacteria^47,48^. Because TDA has been proposed to act as an antiporter^49^, fast-growing bacteria, such as the vibrios^50^, with high metabolic turnover may be more antagonized as compared to slower growing genera of the Bacteriodetes. Ultimately, the increase may have been a result of the indirect effect of the reduction of natural parasites, predators or other species competing for the same niche that are sensitive to TDA in combination with the fact that several Flavobacteriia genera and other members of the Bacteriodetes exhibit natural tolerance to TDA^51,52^. Possible hypotheses for the increase of of Flavobacteria spp. are summarized in Fig. 5.

The *Rhodobacteraceae* comprising the *Phaeobacter* species and other TDA-producing bacteria have previously been suggested to serve as keystone taxa in algal microbial communities^48^. Because of the antagonizing property of TDA, we hypothesized that the WT system could act as an individual stressor and cause perturbation to the system. Indications for perturbed microbiomes have been associated with reduced modularity, decreased microbial diversity and higher positive associations between taxa^28^, however, no difference between the global network properties were observed between the WT and the dTDA network comparisons (Supplementary Table 1).

Node locations varied across the environments and time within both treatments (Fig. 4A; Supplementary Table 1), indicating that the differences in potential microbial interactions were determined by initial pre-colonization of either the WT or the dTDA. Keystone taxa have previously been defined as taxa that exert their influence on microbiome functioning irrespective of abundance as compared to dominant taxa that often affects the functioning of the ecosystem, or a specific process solely by virtue of abundance^53^. While the relative abundance of both WT and dTDA on day 4 was reduced to < 16 %, the WT was simultaneously identified as a keystone OTU along with genera of *Flavobacteriaceae* and *Alteromonadaceae* previously identified as keystone taxa in aquatic ecosystems^48,53^. The fact that the WT was solely identified as a hub in the biofilm, and that the dTDA exhibited no significant network importance suggest that TDA may play a significant role in microbial community assembly during surface colonization.

The WT displayed 1.5 times more positive than negative connections to genera that were highly associated in the microbiome (Fig. 4B). In a recent study by Kehe et al ^13^, growth-promoting positive interactions between metabolic dissimilar genera, and especially between strong and slow-growing bacteria were more common than previously expected^5,11^. Even though the microbial interactions in Kehe et al. ^13^ were mainly governed by carbon source utilization capabilities, our results indicate that the potential of microbial secondary metabolite production, such as TDA, may be linked to potential positive interactions between microbes. Inferring true ecological interactions from network analyses and keystones based on empirically chosen cut-offs should be interpreted with caution as correlation does not equal causation. Hence, bottom-up culture-based studies involving isolates from the same system combined with metabolomics should be used to investigate whether TDA is produced and directly involves positive interactions.

In conclusion, this study suggests that the presence of *Phaeobacter inhibens*, its spatial organization, and its potential to produce TDA can have a considerable impact on the microbial assembly pattern of a marine biofilm. Both differential and network co-occurrence analyses indicated that a large part of the potential interactions between *P. inhibens* and other community members were positive and either indirectly or directly increased the abundances of other species, indicating that TDA may not only be involved in interference competition.

## Materials & methods

### Bacterial strains and growth conditions

The wildtype, *Phaeobacter inhibens* DSM17395^54^ (WT) and the gentamicin-resistant transposon mutant *P. inhibens* DSM17395 *tdaB::GmR* devoid of TDA production^55^ (dTDA) were revived from frozen -80 °C stocks and streaked on Marine Agar (MA, Difco 2216). The GFP-reporter fusion strain, DSM17395-GFP (pBBR1MCS2-PtdaCDE-GFP) and the control strain, DSM17395-NC (pBBR1MCS2) were grown on MA or marine broth (MB) with 100 μg/ml kanamycin (Kan100). Individual colonies were selected and used as biological triplicates of the wildtype and the mutant, respectively, in the following experiments. All cultures were grown at 25 °C in Marine Broth (MB) or on MA.

### Construction of the semi-natural marine model system

Stainless steel coupons (10 × 20 × 1 mm) were cleaned and sterilized as previously described^56^ and clamped vertically in circular stainless steel racks holding up to 20 coupons and placed in 1 L beakers. Single colonies of the WT and dTDA *P. inhibens* were grown in MB overnight. One milliliter of each culture with a mean ± standard deviation (sd) of 8.6 ± 0.04 log^10^ CFU/mL and 8.9 ± 0.15 CFU/mL, respectively, were separately transferred to 250 mL MB in 1 L beakers and allowed to form biofilm on the stainless steel coupons. Non-coated sterile coupons were also immersed in 250 mL MB and used as negative control (Control), and all treatments (3 × 1 L beakers per treatment) were left for three days at 25 °C with agitation (magnetic stirrer; 175 rpm). Coupons with adhered bacteria were gently washed by immersing the circular racks together with coupons twice in 300 mL 3 % Sigma sea salt (Sigma-Aldrich) to remove non-adhering bacteria. Racks and coupons were then submerged in natural seawater collected in Hellerup harbor, Denmark (55.7319, 12.5810), on September 24, 2018 at 10.45 a.m. at approximately 0.1-meter depth (13.5 °C, 3.32 mg L^-1^ dissolved oxygen [DO], 24.90 [PSU]). The total number of bacterial cells in seawater was determined using SYBR Gold staining and counting by epifluorescence microscopy^57^. The racks with biofilm-coated coupons and the non-treated Control were separately incubated with 250 mL seawater at 25 °C with agitation (magnetic stirrer; 175 rpm) for 10 days. Bacteria in the biofilm were harvested by transferring single coupons to polystyrene tubes containing 2 mL 3 % SSS. Tubes were sonicated for 4 min at 25 °C (28-kHz, 2 × 150 W sonication bath, Delta 220, Deltasonic, Meaux, France) and vortexed at maximum speed for 15 s to detach bacteria from the surfaces^58^. The bacteria detached from the biofilm and 2 mL of the planktonic suspension were centrifuged at 8,000 × *g* for 20 min and the pellet was then used for DNA extractions. Samples were collected from all treatments (in three biological replicates) at day 0, 1, 4, and 10 in three technical replicates per beaker.

### Construction of a DSM17395 GFP reporter fusion strain

The promoter region of the *tdaCDE* operon was amplified using the primer pairs PtdaB-Pinh_Fw (5′-AAAACTCGAGCTGGTTTGAGCGCTGCCT-3′) and PtdaC-gfp-Rv (5′-TTCTCCTTTACGCATGGCCTGTTCCATCGTCC-3′). The promoter-less gfpmut3* open reading frame was amplified using the primer pair GFPmut3-Fw (5′-ACGATGGAACAGGCCATGCGTAAAGGAGAAGAACTTTTC-3′) and GFPmut3-Rv (5′-ATCCCGGGTTATTTGTATAGTTCATCCATGC -3′) and the PCR products were fused to the *tdaCDE* promoter segment via overlap PCR with primers PtdaB-Pinh and GFPmut3-Rv. The Q5 hot start high fidelity 2 × master mix (New England Biolabs, M0494S, BioNordika, Denmark) was used for the reactions. The fused PtdaCDE-gfp segments were cloned between the XhoI and XmaI sites of the pBBR1-MCS2 vector^59^ *via* restriction digestion cloning using the Quick Ligation Kit (New England Biolabs, M2200, BioNordika, Denmark), which resulted in plasmid pBBR1-MCS2-PtdaCDE-gfp. The recombined plasmid, as well as the pBBRMCS2 empty vector, were transferred into strain *Escherichia coli* WM3064, which was subsequently used as donors to conjugate the plasmids into the wild type DSM17395 strain^60^. This resulted in the strains DSM17395-GFP and DSM17395-NC.

### Expression from the *tdaCDE*-promoter in an axenic model system

Expression from the *tdaCDE* promoter was determined in the pre-formed biofilm on the stainless steel coupons of either the DSM17395-GFP or the DSM17395-NC strains. Both strains were incubated for 3 days in 250 mL MB supplemented with Kan100 together with clean coupons in parallel beaker systems as described above. Subsequently, racks and coupons were transferred to 1-L beakers with 250 mL 2.4 % instant ocean (IO) and incubated for 10 days. Green fluorescence was detected from the attached *P. inhibens* cells after 0, 1, 4, and 10 days. Coupons were washed three times with 2.4 % IO to remove planktonic cells and placed on a cover slide. GFP-fluorescence images were captured using a 60× oil objective on a Nikon Inverted Fluorescence Microscope-EclipseTi2 (Nikon, Tokyo, Japan; WIB ex. 457–487 nm, em. 502–537 nm). The NIS-Element software was used for the acquisition of representative fluorescence micrographs with an exposure time of 170 ms. A total of 2 µL of a DAPI stain solution (of 100 ng/µL) was applied to the same specimen and fluorescence of DAPI (WIB ex. 352–402 nm, em. 417–477 nm) was recorded right after the staining. Images were processed using the Fiji package from ImageJ^61^ and any adjustments were made to the entire image and equally across samples.

### DNA extractions

Cell pellets were lysed in 400 µL lysis buffer (400 mM NaCl, 750 mM sucrose, 20 mM EDTA, and 50 mM Tris-HCl [pH 8.3]) with a final concentration of 1 mg/mL lysozyme (Sigma, St. Louis, MO, USA). Cell lysates were incubated at 37 °C for 30 min, and sodium dodecyl sulfate (SDS) and proteinase K (Sigma, St. Louis, MO, USA) were added to final concentrations of 1 % and 0.1 mg/mL, respectively. After overnight incubation at 55 °C with regular agitation, samples were centrifuged at 3000 × *g* for 5 min and the supernatants were transferred to the lysate chamber in the Maxwell® 16 LEV Blood DNA Kit (Promega, Madison, WI, USA) and processed with the Maxwell™ 16 instrument. Finally, DNA was diluted into 50 µL TE elution buffer provided in the Maxwell® 16 LEV Blood DNA Kit.

### Quantification of bacterial absolute abundances and *P. inhibens*

The absolute bacterial and *P. inhibens* abundances in the biofilm and the planktonic suspension samples were quantified by qPCR using the 16 S rRNA gene universal primers (338 F/518 R), as described in Bernbom et al ^62^, and *Phaeobacter* spp. specific primers (Pi_Fw/Pi_Rev) developed by Dittmann et al. ^47^. Standard curves relating C_*T*_ to CFU/mL were based on gDNA extracted from five tenfold serial dilutions of *Phaeobacter* strain 27-4^21^, *Pseudoalteromonas tunicata* D2^63^, and *Vibrio anguillarum* 90-11-287^64^ for the estimation of total bacterial abundance and *P. inhibens* DSM17395 for the estimation of *Phaeobacter* spp. abundances. Each qPCR reaction contained 7.5 µL PowerUp™ SYBR® Green 2× Master Mix (ThermoFischer; A25742), 1.05 µL of forward (338 F; ACT CCT ACG GGA GGC AGC AG, Pi_Fw; 5′GTG TGT TGC GGT CTT TCA CC′3) and reverse (518 R; ATT ACC GCG GCT GCT GG, Pi_Rev; 5′AGG ACC ATG TCC CCT CTA CC′3) primer (10 µM), 4.4 μL DNAse free water and 1 μL DNA template. Standards and samples were run in duplicates on each plate. The MX3000P instrument (Stratagene, La Jolla, CA) was used with the following cycling conditions: 50 °C for 2 min, 95 °C for 2 min [95 °C for 15 s, 60 °C for 1 min] × 40 cycles.

### Amplicon sequencing of the 16 S rRNA V3-V4 region

The V3-V4 region was amplified across all samples, including genomic DNA from *P. inhibens* DSM17395, using PCR-primers (Fw_V3-V4: 5′-CCTACGGGNGGCWGCAG-3′ and Rv_V3-V4: 5′-GACTACHVGGGTATCTAATCC-3′) tagged with octameric barcodes, amplified, cleaned and pooled in equimolar ratios as described in Bech et al. (2020)^65^. Amplicons were sequenced by Novogene, Cambridge, United Kingdom on the Illumina NovaSeq 6000 250PE platform.

### V3-V4 amplicon processing with QIIME2 and DADA2

The forward and reverse sequences were processed in the QIIME2^66^ pipeline, v2020.8^66^. The 8 nt barcodes were removed using the qiime cutadapt demux-paired plugin. Later, paired-end reads were subjected to quality control followed by denoising, merging, and chimera removal using the DADA2 plugin implemented in QIIME 2 (dada2 denoise-paired with the following parameters trunc_len_f:242, trunc_len_r:242). The final amplicon sequence variant (ASV) table and the corresponding denoised ASVs were imported and analyzed in R. Denoised ASVs were individually taxonomically classified using the RDP naive Bayesian classifier^67^ trained with the SILVA v132 16 S rRNA gene sequence database^68^. A total of 50,223,017 reads passed quality control, with a mean ± sd of 250,017 ± 201,619 reads per sample, where the rarefaction curves of the ASV diversity was fully saturated across all samples (https://github.com/PKBech/PRJNA795304)). All ASVs were aggregated to genus level and low abundant genera (<100 reads across all samples) were removed from the dataset (0.0028 % of the total reads) leaving a total of 1056 genera in the final genus table (Table 1). Two ASVs were identified from the V3-V4 region amplicon sequencing of the genomic DNA of *P. inhibens* DSM17395. Since the V3-V4 region of this species is uniquely identifiable^69^, the sum of relative abundances of the two specific ASVs were added together as a single OTU referred to as the ‘*P. inhibens* OTU’ in the genus table.

### Beta-diversity

Prior to beta-diversity analysis, the *P. inhibens* OTU was excluded from the genus table, normalized and scaled to a total sum of 100,000 reads per sample. All the analyses were performed using the ‘vegan’ R package v2.5 (see ‘URLs’ section). Bray-Curtis dissimilarities were calculated from the square-root transformed genus table used to determine the variation in the microbial community composition explained by the three categorical variables: time (day 1, 4, and 10), treatment (Control, WT- and dTDA systems) and environment (biofilm and planktonic). First, we verified that the beta-dispersion was similar and non-significantly different (*p-*value > 0.05) between the groups across all samples by the betadisper function. Next, the variance explained by the three independent variables was tested using a permutational multivariate analysis of variance (PERMANOVA) with the adonis function. As a *posthoc* test for pairwise differences within each variable, the pairwise.adonis2 function (see ‘URLs’ section) was used to specifically compare differences between the WT and the dTDA groups. The results were visualized by non-metric multidimensional scaling (nMDS) ordination plots using the metaMDS function with *k* = 3, since three dimensions achieved a stress value < 0.1.

### Statistical analyses on alpha-diversity and absolute abundances

The observed richness was calculated by the estimateR function from the normalized genus table, including the *P. inhibens* OTU and analyzed with linear mixed-effects models (LMM) using the biological replicate as random effects with the lme4 package^70^. Differences in the marginal means (EMM) of the linear models of the observed richness and the absolute abundances of the total microbial population and *Phaeobacter* spp. quantified by qPCR at the individual days were estimated by the emmeans function in the ‘emmeans’ package (see ‘URLs’ section). Differences between seawater samples collected at day 0 and all other samples were tested by the Dunnest test. Means and standard deviations were summarized in Table 1.

### Differential relative abundance analyses

We used the ANCOM-BC model, which is optimized to cope with and minimize the challenges associated with the nature of microbiome compositional data^71^, to identify differentially abundant genera and orders that responded to the pre-colonization of the *Phaeobacter* strains. The sum of differentially abundant genera was identified between the WT and the dTDA system by each day and environment. The non-normalized genus table excluding the *P. inhibens* OTU for each day and environment (*N* = 8–9) were analyzed with the ancombc function with default parameters and a cut-off of Holm adjusted *p*-values < 0.01 with the WT treated samples set as comparing reference group. The sum of differentially abundant genera found by each comparison were summarized in a balloonplot using the ggballoonplot function from the ggpubr R package (see ‘URLs’ section). To get the taxonomic profile of the log-fold differential changes by each comparison, all samples from day 4 were aggregated to order level and analyzed as described above for both biofilm and the planktonic suspension.

### Co-occurrence networks

We constructed different networks to infer keystone taxa in the WT and the dTDA systems, using the NetCoMi package^72^ developed to compare and quantify network differences between co-occurrences of different genera and the *P. inhibens* OTU. A total of six comparison networks were constructed with the WT and the dTDA systems by day and environment. The SparCC method was used as the correlation method, which is developed for inferring correlations for compositional data and has been shown to be efficient in detecting co-occurrences in communities exhibiting relatively low Simpson diversity^73^. Genera having reads >100 in more than 5 replicates and a sparsity threshold of 0.6 were used to obtain the correlation matrix. The netAnalyze function was used for network characterization with the cluster_fast_greedy function to identify clusters and modularity. The scores of betweenness, degree, closeness, and eigenvector centrality have previously been used to capture different aspects of node importance and statistically identify keystone genera^74–76^. In this study, we defined hubs—or ‘keystone OTUs’ with the highest eigenvector centrality value above the empirical 95 % quantile^72^, combined by high degree, high closeness and low betweenness centrality, which have previously been suggested to be indications for keystone OTUs^77^. The plot.microNetProps function was used to visualize the network and quantitative network comparisons were done with the netCompare function using 1000 permutations.

### URLs

Vegan package v2.5: https://github.com/vegandevs/vegan. Pairwise.adonis2 function: https://github.com/pmartinezarbizu/pairwiseAdonis. The emmeans function in the ‘emmeans’ package: https://github.com/rvlenth/emmeans. The ggballoonplot function from the ggpubr R package: https://rpkgs.datanovia.com/ggpubr/.

## Supplementary information

Supplementary material

Supplementary Table 1

## Data Availability

Sequencing data is available in the Sequencing Read Archive (SRA), amplicon data in BioProject PRJNA795304 and R scripts at https://github.com/PKBech/PRJNA795304. The source data underlying Figs. 1–4 and Supplementary Figs. 2-3 are provided as a Source Data file.

## Source data

Source data
