# Supplementary material for: The potential to produce tropodithietic acid by Phaeobacter inhibens affects the assembly of microbial biofilm communities in natural seawater
Source: NPJ Biofilms Microbiomes. 2023 Mar 23;9:12. doi: 10.1038/s41522-023-00379-3 (PMC10036634; doi:10.1038/s41522-023-00379-3)
Supplement: Supplementary file 1 — Supplementary material [file 41522_2023_379_MOESM1_ESM.pdf]

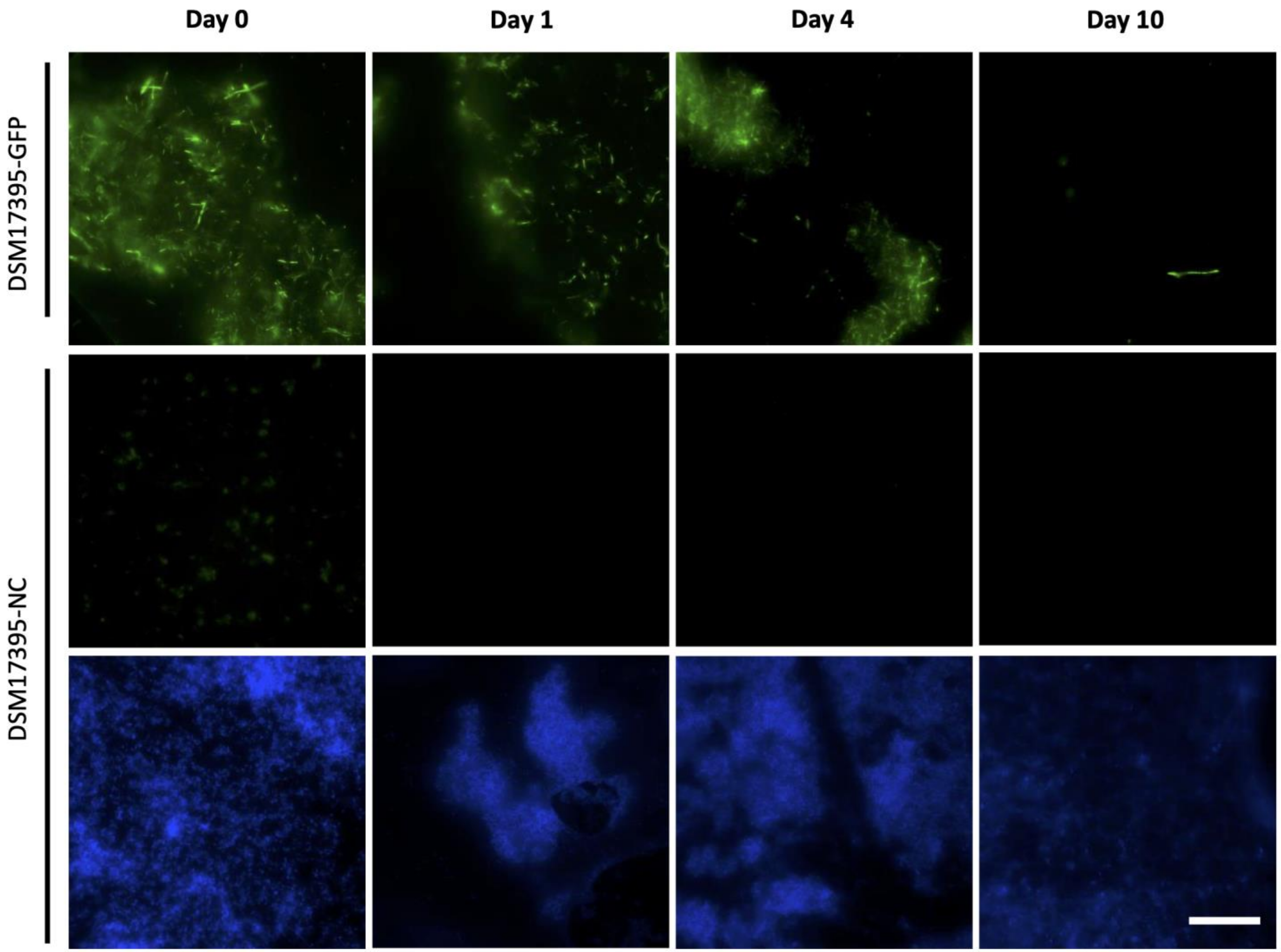

Day 0
Day 1
Day 4
Day 10
DSM17395-GFP
DSM17395-NC

Supplementary Figure 1: Transcription activity of the *tdaCDE* promoter indicated by the GFP fluorescence was detected on the pre-coated surface with *P. inhibens* DSM17395-GFP in the axenic model system. At day 0, pre-coated stainless steel coupons were transferred from cultures in MB into 2.4% IO beaker systems. After 0, 1, 4 and 10 days, fluorescence micrographs of cells attached to the coupons were captured. The GFP fluorescence indicates transcription from the *tdaCDE* promotor. Pre-coated coupons with the negative control (DSM17395-NC) were also captured by DAPI staining (lower panel). Scaled bar indicates 50 µm.

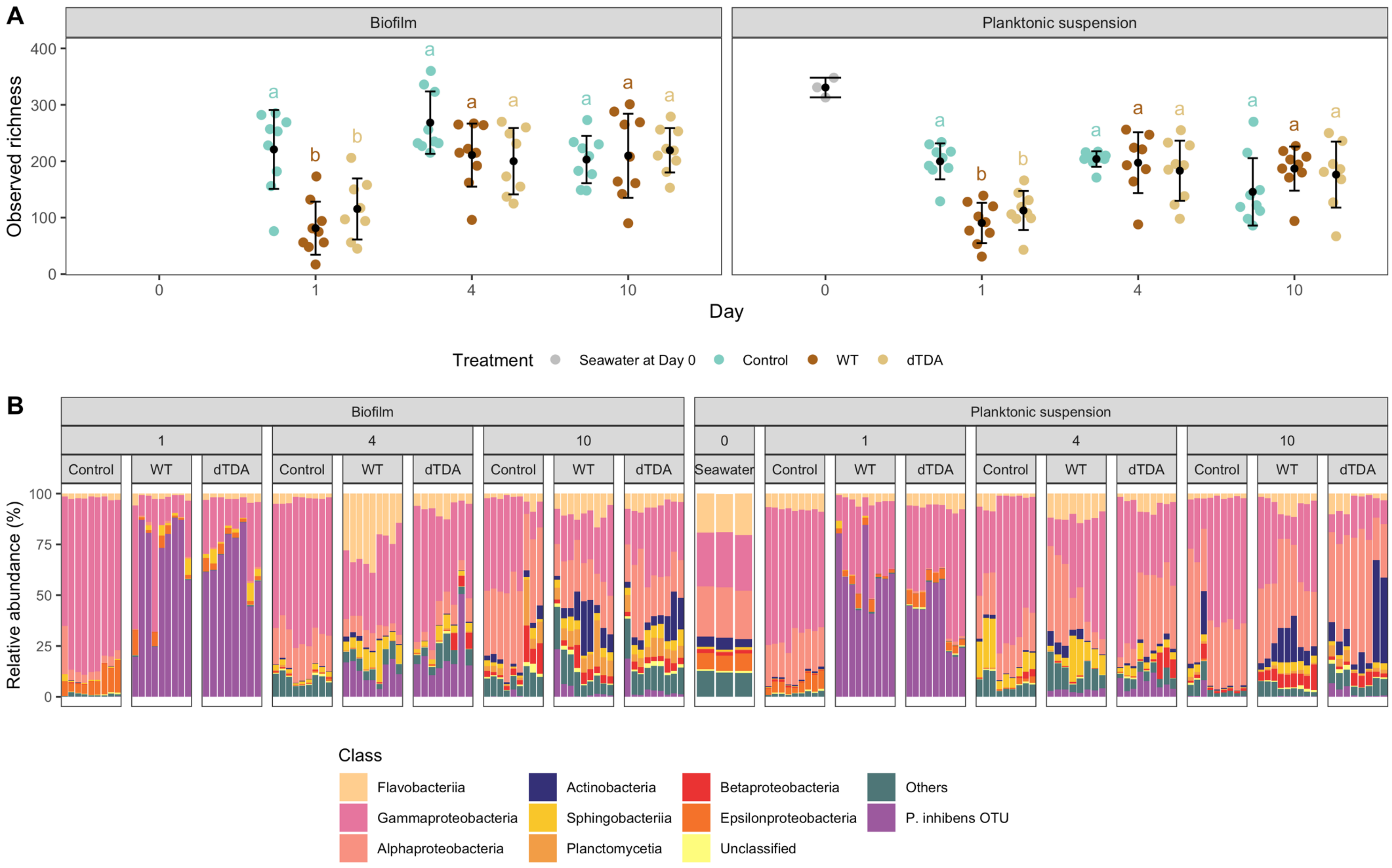
A
Biofilm
Planktonic suspension
Observed richness
Day
Treatment
Seawater at Day 0
Control
WT
dTDA
B
Relative abundance (%)
Seawater
Class
Flavobacteriia
Gammaproteobacteria
Alphaproteobacteria
Actinobacteria
Sphingobacteriia
Planctomycetia
Betaproteobacteria
Epsilonproteobacteria
Unclassified
Others
P. inhibens OTU

Supplementary Figure 2: The alpha diversity and microbial composition across all in the tree systems (Control, WT and dTDA). **A:** The observed richness; in black are means (dots) and standard deviation (error bars) and significance letters (LMM & EMM; *p*-value < 0.05) between the treatments per day. **B:** The relative abundances of *P. inhibens* OTU and the microbial composition at class level. Source data are provided as a Source Data file.

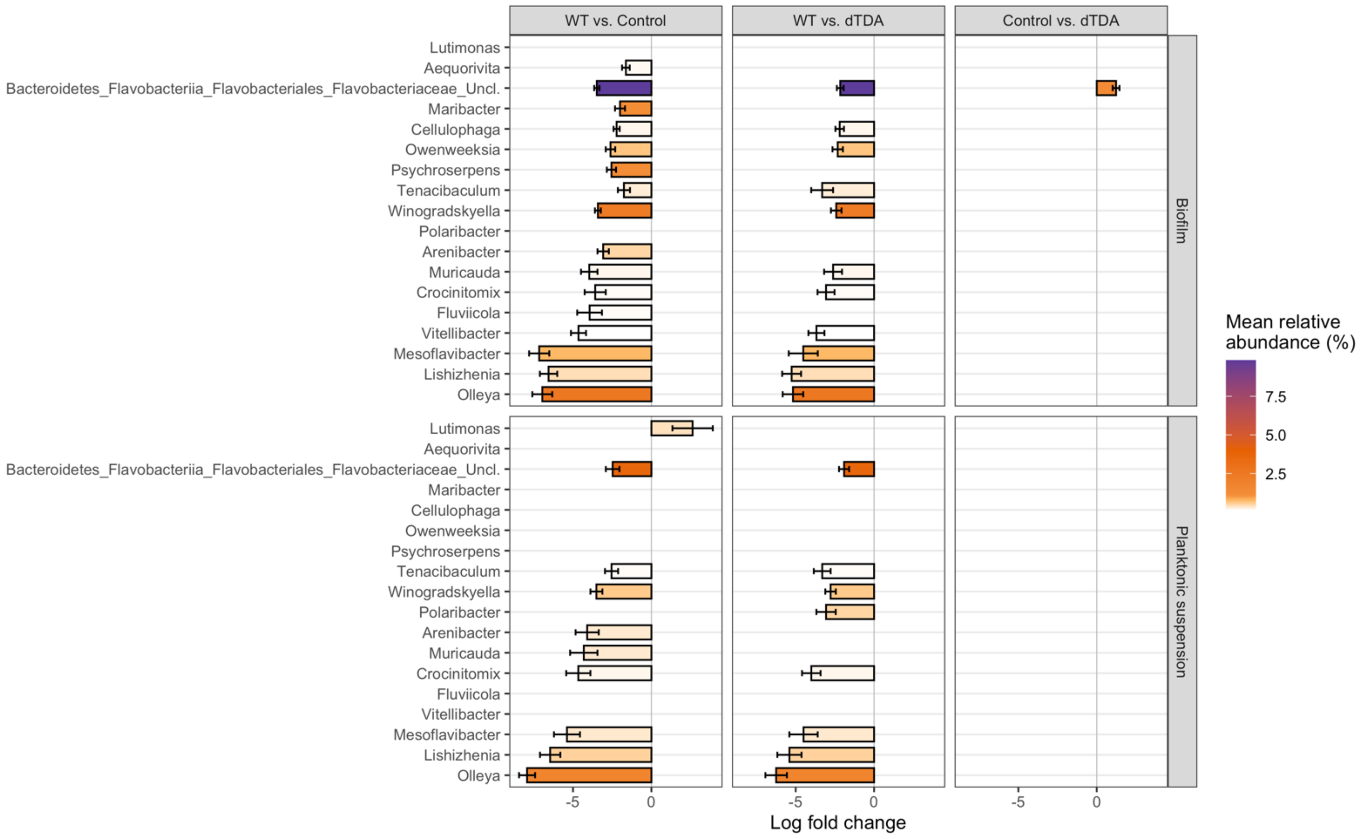

Supplementary Figure 3: Identification of genera of the *Flavobacteriales* that are differentially abundant between the WT and the dTDA, WT and the Control and the dTDA and Control systems identified by the ANCOM-BC model using a 95% confidence interval (two-sided; Holm adjusted) and adjusted *p*-values <0.05 stratified by time and environment. ASVs are aggregated to genus level and represented by effect size

(Log^{10} fold change). Bars are coloured according to the mean relative abundance of the order enriched by the treatment relative to the other. Source data are provided as a Source Data file.
